# Identification and Validation of a Potent Multi-lncRNA Molecular Model for Predicting Gastric Cancer Prognosis

**DOI:** 10.3389/fgene.2021.607748

**Published:** 2021-12-20

**Authors:** Zhiguo Guo, Erbo Liang, Tao Zhang, Mengqing Xu, Xiaohan Jiang, Fachao Zhi

**Affiliations:** ^1^ Guangdong Provincial Key Laboratory of Gastroenterology, Institute of Gastroenterology of Guangdong Province, Department of Gastroenterology, Nanfang Hospital, Southern Medical University, Guangzhou, China; ^2^ Department of Gastroenterology, Suzhou Hospital of Anhui Medical University, Suzhou, China; ^3^ Department of Gastrointestinal Surgery, Suzhou Hospital of Anhui Medical University, Suzhou, China

**Keywords:** gastric cancer, long noncoding RNA, overall survival, competing endogenous RNA network, risk prediction model

## Abstract

Gastric cancer (GC) remains the third deadliest malignancy in China. Despite the current understanding that the long noncoding RNAs (lncRNAs) play a pivotal function in the growth and progression of cancer, their prognostic value in GC remains unclear. Therefore, we aimed to construct a polymolecular prediction model by employing a competing endogenous RNA (ceRNA) network signature obtained by integrated bioinformatics analysis to evaluate patient prognosis in GC. Overall, 1,464 mRNAs, 14,376 lncRNAs, and 73 microRNAs (miRNAs) were found to be differentially expressed in GC. Gene Ontology (GO) function and Kyoto Encyclopedia of Genes and Genomes (KEGG) pathway analyses revealed that these differentially expressed RNAs were mostly enriched in neuroactive ligand–receptor interaction, chemical carcinogenesis, epidermis development, and digestion, which were correlated with GC. A ceRNA network consisting of four lncRNAs, 21 miRNAs, and 12 mRNAs were constructed. We identified four lncRNAs (lnc00473, H19, AC079160.1, and AC093866.1) as prognostic biomarkers, and their levels were quantified by qRT-PCR in cancer and adjacent noncancerous tissue specimens. Univariable and multivariable Cox regression analyses suggested statistically significant differences in age, stage, radiotherapy, and risk score groups, which were independent predictors of prognosis. A risk prediction model was created to test whether lncRNAs could be used as an independent risk predictor of GC or not. These novel lncRNAs’ signature independently predicted overall survival in GC (*p* < 0.001). Taken together, this study identified a ceRNA and protein–protein interaction networks that significantly affect GC, which could be valuable for GC diagnosis and therapy.

## 1 Introduction

According to global cancer statistics, gastric cancer (GC) represents a major fatal tumor, with an incidence rate of 5.7% and a mortality rate of 8.2% (Bray et al., 2018). GC remains a life-threatening disease among Chinese individuals due to its high malignancy, low survival rate, and poor prognosis (Chen et al., 2016; Allemani et al., 2018; Bray et al., 2018). Despite recent technical and medical advances, overall survival (OS) in GC remains low, owing to its detection at the advanced stages of proliferation. Therefore, early diagnosis is of great significance in ameliorating patient prognosis in GC (Tan, 2019).

Long noncoding RNAs (lncRNAs) are a class of new and potent tumor regulator RNAs, with >200 nucleotides and low protein-coding potential (Guttman et al., 2009; Ponting et al., 2009). LncRNAs have critical regulatory functions in multiple biological events, such as cell cycle, differentiation, proliferation, metastasis, apoptosis, and invasion in various tumor cells. Moreover, lncRNAs are intimately related to etiological events in various diseases and cancers (Batista and Chang, 2013; Zhang and Song, 2018). For example, lnc00473 significantly affects tumor promotion in multiple cancers, with obvious roles in GC cell growth, adhesion, and migration. High expression of the lncRNA H19 can promote GC occurrence and metastasis, while p53 may determine H19 elevation in hypoxic cancer cells (Matouk et al., 2010; Zhou et al., 2015a). Due to high specificity, accessibility, noninvasive detection, and abnormal expression under different pathophysiological conditions, lncRNAs are considered to be a potential marker for GC diagnosis, prognosis, and potential therapeutic target in this malignancy.

In recent years, competing endogenous RNAs (ceRNAs) have enticed the attention of researchers to further examine the molecular mechanisms of cancer occurrence and development. ceRNAs actually compete with mRNA molecules for the same pool of miRNAs. It is reported that miRNAs regulate mRNAs by interacting with their 3ʹ-UTRs. However, lncRNAs compete with these miRNAs to control gene expression (Cao et al., 2018; El-Sakka et al., 2018). Recently, it was suggested that lncRNAs in combination with mRNAs may improve cancer diagnosis, although limited reports have assessed the ceRNA network in GC (Salmena et al., 2011). Hence, the regulatory functions of lncRNA as ceRNA in GC are still ambiguous.

Guan et al. (2019) determined four hub genes (*MCM4, KIF23, MCM8*, and *NCAPD2*) by establishing a protein–protein interaction (PPI) network in GC. Liu et al. (2018) also confirmed that the lncRNAs *DLEU2* and *DDX11* were significantly upregulated in GC tissues, in which DLEU2 promotes cell proliferation, whereas DDX11 negatively regulates miRNA expression. Furthermore, *DLEU2* and *DDX11* also act as potential ceRNAs to sponge miRNAs. In another study pertaining to ceRNA network analysis, Arun et al. (2018) revealed that miR-21 and miR-148a act as central candidates synchronizing the decoying activities of the lncRNAs (H19, TUG1, and MALAT1). They also proposed that overexpression of H19 and miR-21 are characteristic features of gastric tumorigenesis, which makes them ideal prognostic indicators and potential therapeutic targets.

HOXC-AS3 binds to YBX1, to transcriptionally regulate a large set of genes that contribute to the proliferation and migration of GC cells, such as MMP7, WNT10B, and HDAC5 (Zhang et al.). Another study showed that KRT19P3 exerts tumor-suppressive effects in GC by directly interacting with COPS7 and, thereby, leading to the suppression of cell proliferation, migration, and invasion (Zheng et al.); thus, KRT19P3 s associated with poor prognosis in patients with GC.

Here, we evaluated the non-coding RNA landscape in GC and established a multi-molecular prognosis model of lncRNAs for OS prediction of patients with GC. Furthermore, the functions of the identified ceRNAs were predicted to provide insights into the associated molecular mechanisms and thereby potentially assist in developing novel therapeutic targets against GC.

## 2 Materials and Methods

### 2.1 Human Tissue Samples

We collected GC and nonmalignant tissue (adjacent to GC) samples from eight patients being treated at Suzhou Hospital of Anhui Medical University, Suzhou, China. Prior to surgical resection, all patients had been diagnosed with GC pathologically, with no previous chemotherapy or radiotherapy. The detailed clinical information of these patients is summarized in Supplementary Table S1. This study was approved by the Ethics Committee of Anhui Medical University (20200663), and each patient provided informed consent.

### 2.2 Patient Datasets

RNA sequencing (RNAseq) and miRNAseq count data of patients with gastric adenocarcinoma, including 375 patients and 32 control samples in RNAseq, and 436 patients and 41 control samples in miRNAseq, were retrieved from The Cancer Genome Atlas database (https://www.cancer.gov/about-nci/organization/ccg/research/structural-genomics/tcga). The corresponding clinical information of gastric adenocarcinoma patients was retrieved from UCSC XENA (https://xenabrowser.net/datapages/). The R language package *DEseq2* was used to detect differential expression. The threshold was set at |logFC| > 2 and false discovery rate (FDR) < 0.01 for screening and acquiring the final fold change differentially expressed mRNAs, lncRNAs, and miRNAs.

### 2.3 LncRNA Data Screening

Firstly, the grouping matrix was constructed based on the expression matrix of RNAseq and miRNAseq after a trimmed mean of M-values normalization. The grouping matrix contained the grouping information of samples to provide differential expression analysis. Then, a differential comparison matrix was constructed to specify sample pairs to be compared mutually for downstream analysis, i.e., cancer and normal samples in this study. It should be noted that the current RNAseq count file generally includes not only regular mRNAseq data, but also lncRNA data. Therefore, lncRNAs included “3prime_overlapping_ncRNA,” “antisense_RNA,” “bidirectional_promoter_lncRNA,” “lincRNA,” “macro_lncRNA,” “non_coding,” “processed_transcript,” “sense_intronic,” and “sense overlapping”.

### 2.4 Functional Enrichment Analysis

For the comprehensive analysis of the cellular functions of the differentially expressed RNAs (DERNAs) in the ceRNA network, we performed GO and KEGG pathway enrichment analyses (Supplementary Table S2) using DAVID v6.8 (https://david.ncifcrf.gov/) and KOBAS 3.0 (http://kobas.cbi.pku.edu.cn/kobas3/genelist/), with an FDR <0.05 being considered statistically significant. A PPI network was established with coexpressed genes having 95% confidence interval (CI) >0.9 in String (v11.0, https://string-db.org), and was visualized using Cytoscape 3.8.0 software.

### 2.5 LncRNA–mRNA–miRNA ceRNA Network Construction

**FIGURE 1:**
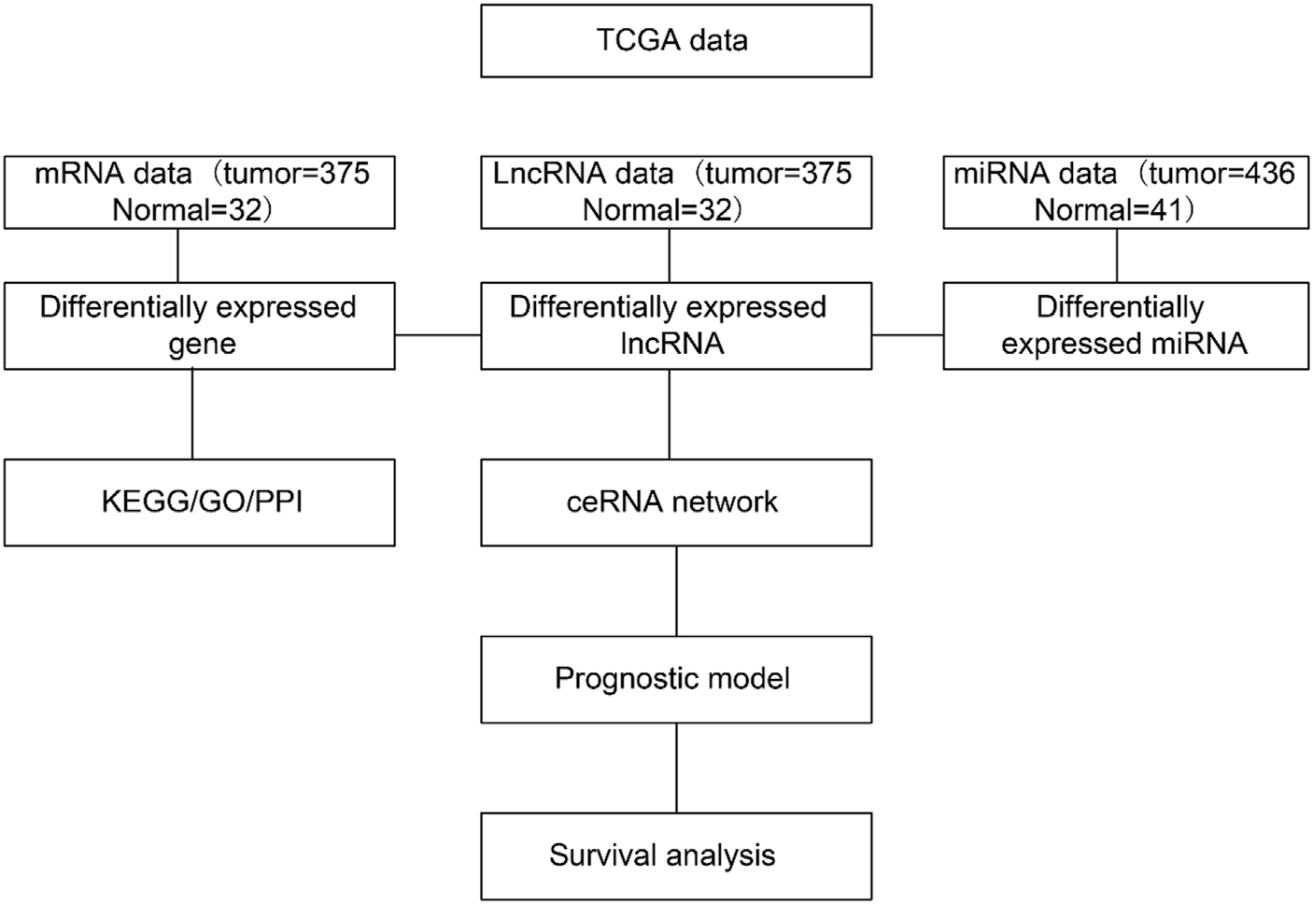
Bioinformatics pipeline to construct the competitive endogenous RNA (ceRNA) network. DERNA, differentially expressed RNA; lncRNA, long noncoding RNA; miRNA, micro RNA; mRNA, messenger RNA.

The DERNAs were used to construct a lncRNA–mRNA–miRNA ceRNA network using the GDC RNA Analysis function. Three criteria were used to determine the endogenous competition effect of the mutual competition of lncRNA–mRNA: the lncRNA and mRNA must share a significant number of miRNAs; the lncRNA should be positively correlated with the mRNA; and the common miRNA should play a similar role in modulating the lncRNA and mRNA (Figure 1 **)**.

### 2.6 Risk Score Establishment

To verify the significance of the lncRNAs, a risk score was developed for establishing a comprehensive signature for prognostic assessment. The following equation was used to calculate the risk score:

*Risk score* = (0.0299*expression value of H19) + (0.0734*expression value of LINC00473) + (0.0732*expression value of AC079160.1) + (−0.0025*expression value of AC093866.1).

Each sample was scored by linearly combining the expression of the lncRNAs. For the subsequent assessment, the specimens were divided into low–risk and high–risk groups. The cutoff value was defined based on the median risk score value to compare the survival time.

### 2.7 Gene Expression Omnibus Datasets

To validate the prognostic value of the designed model, a GC-related microarray dataset (GSE62254), comprising 300 GC samples, was retrieved from the GEO database (http://www.ncbi.nlm.nih.gov/geo). The extracted data were normalized and processed by log2 transformation. The preprocess *Core* R package was used to normalize the microarray data, the *limma* R package was then used for differential expression analysis, and the *survival*, *survivalROC*, *survminer*, and *pheatmap* R packages were performed for survival analysis.

### 2.8 Quantitative Real-Time Polymerase Chain Reaction (qRT-PCR)

Total RNA was extracted from collected tissues and each cell line using TRIzol Reagent (Invitrogen, United States) according to the manufacturer’s instructions. Then, SuperScript™ III First-Strand Synthesis SuperMix qRT-PCR (Takara) was used for reverse transcription reaction; three repeats of qRT-PCR were performed using Power SYBR^®^ Green PCR Master Mix (Applied Biosystems) and CFX384 Real-Time PCR detection system (Bio-RAD, United States). The following qRT-PCR condition was used: 95°C 60 s, 95°C 15 s, and 63°C 25 s for 40 cycles. Human GAPDH was used as an internal control and was analyzed by the 2^−ΔΔCT^ method. The primers were purchased from Shanghai Sangong Bioengineering Co., Ltd. A list of primers is shown in Supplementary Table S3.

### 2.9 Statistical Analysis

SPSS 24.0 (IBM Corp., United States) and Prism 8 (GraphPad Software, United States) were used for statistical analyses. The data are represented as mean ± standard deviation (unless otherwise stated). Student’s *t*-test was used to compare group pairs. For comparing the prognostic values, the univariate and multivariate Cox regression analyses were performed, with the risk score as a categorical factor along with other clinical variables. Additional bioinformatics analyses were performed in R. *p* < 0.05 indicated statistical significance.

## 3 Results

### 3.1 Identification of Differentially Expressed lncRNAs, miRNAs, and mRNAs in GC

**FIGURE 2:**
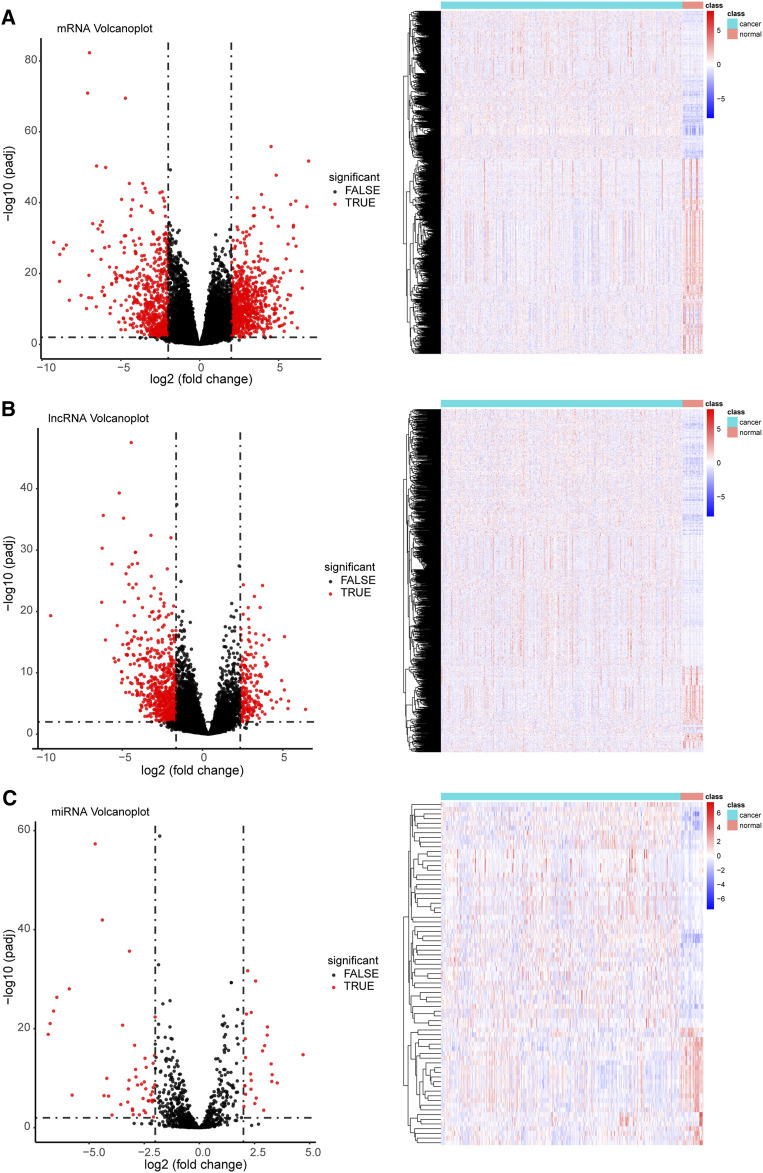
Coexpression profile of differentially expressed target genes (volcano plots and heatmaps) among cancer and normal tissue samples. **(A)** A total of 1,464 differentially expressed mRNAs (826 upregulated and 638 downregulated), **(B)** 910 differentially expressed lncRNAs (206 upregulated and 704 downregulated), and **(C)** 73 differentially expressed miRNAs (25 upregulated and 48 downregulated) were identified (threshold set at |log (Fold Change) > 2| and with a false discovery rate < 0.01). For heatmaps, the colors red and blue indicate high and low relative expression, respectively. For volcano plots, red color on the right and left indicate upregulated and downregulated genes, respectively.

Overall, 1,464 mRNAs, 14,376 lncRNAs, and 73 miRNAs with differential expression were identified in GC **(** Figure 2 and Supplementary Table S4). Of the 1,464 differentially expressed mRNAs, 826 were upregulated, whereas 638 were downregulated. In the case of lncRNAs, 206 and 704 were upregulated and downregulated, respectively. However, the miRNAs displayed the lowest numbers of differentially expressed genes, with only 25 upregulated and 48 downregulated genes in comparison to mRNAs and lncRNAs. The co-expressed DERNAs are depicted in volcano plots and heatmaps in Figure 2.

### 3.2 Functional Prediction of the lncRNA–miRNA–mRNA ceRNA Network

**FIGURE 3:**
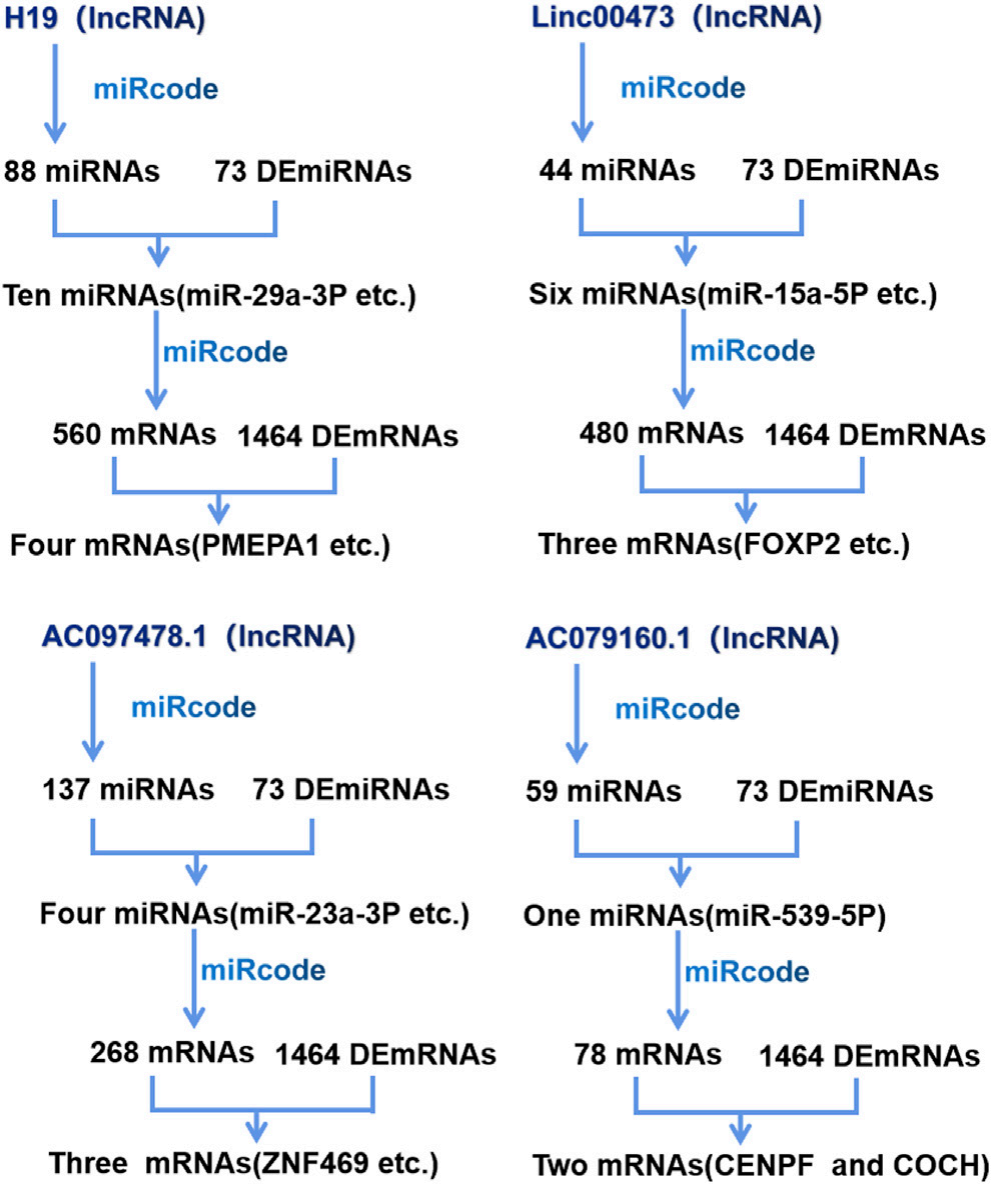
Construction of the competitive endogenous RNA (ceRNA) network. DERNA, differentially expressed RNA; lncRNA, long noncoding RNA; miRNA, micro RNA; mRNA, messenger RNA.

By utilizing the existing knowledge that lncRNAs modulate mRNA expression by serving as a miRNA decoy, GC-specific lncRNAs, miRNAs, and mRNAs were used to build a ceRNA network (Figure 3). The lncRNA–miRNA interactions were based on the miRcode algorithm (http://www.mircode.org/) considering the GC-specific miRNAs. Additionally, Targetscan (http://www.targetscan.org/), miRdb (http://www.mirdb.org/), and miRTarBase (http://mirtarbase.mbc. nctu.edu.tw/) were used to identify miRNA-targeted mRNA. Intersections between the differentially expressed lncRNAs, miRNA, and mRNAs were identified and used to construct the lncRNA–miRNA–mRNA ceRNA network (Figure 3) using Cytoscape. The lncRNAs involved in the network were designated “key lncRNAs.”

**FIGURE 4:**
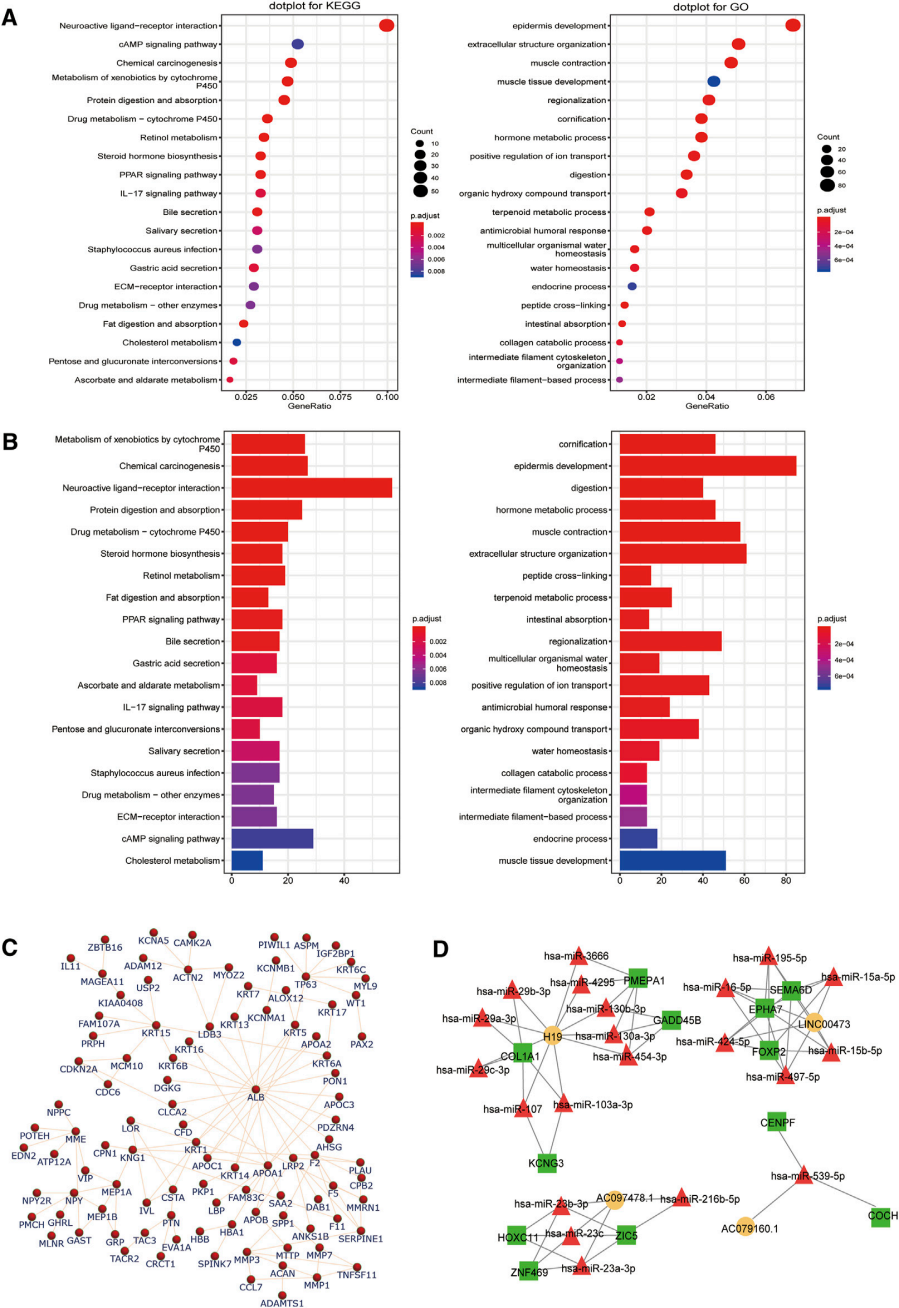
Identification of key pathways and proteins related to the differentially expressed long noncoding RNAs (lncRNAs) and messenger RNAs (mRNAs) involved in the competitive endogenous RNA (ceRNA) network. Top 20 enriched Kyoto Encyclopedia of Genes and Genomes (KEGG) pathway and gene ontology (GO) enrichment analysis of the differentially expressed **(A)** lncRNAs and **(B)** mRNAs. The count represents the genes related to the GO term in KEGG or biological processes. **(C)** In the network diagram, the genes are depicted by the nodes while the interactions are shown by the edges. These protein–protein interactions are based on mRNAs, and the network was constructed using the STRING tool. **(D)** The lncRNA–microRNA–mRNA network was designed using the correlation analysis between all the DERNAs. Legend: square, mRNA; circle, lncRNA; triangle, miRNA. Orange, red, and green symbols represent upregulated lncRNAs, downregulated miRNAs, and upregulated mRNAs, respectively.

To predict the function of the differentially expressed genes, GO, KEGG enrichment, and PPI network analyses were performed using online tools. The top 20 identified GO terms are described in Figure 4A. Functional analysis by KEGG revealed the top 10 significantly enriched pathways, including neuroactive ligand–receptor interaction, chemical carcinogenesis, and epidermis development and digestion (Figure 4B). Next, 590 differently expressed mRNAs were imported into the STRING tool to establish their PPI network and determine the functional relationships among them (Figure 4C). Several proteins (ALB, TP63, ACTN2, KRT15, MMP3, PTN, and NPY, among others) were identified as star molecules of the network, with a particular focus on ALB that interacted with 21 proteins within the network.

### 3.3 CeRNA Network in GC

For further assessing the roles of lncRNAs in GC and elucidating the interactions among different RNAs, a ceRNA network analysis was performed using lncRNA–miRNA–mRNA data in the GDC RNA tool. Moreover, the mutual competitions of lncRNA–miRNA and mRNA–miRNA were identified according to information from the miRcode database, which was further used to predict the functions of the lncRNAs. Finally, Cytoscape was used to visualize the network (Figure 4D).

### 3.4 Identification and Validation of the Prognostic Signature

**FIGURE 5:**
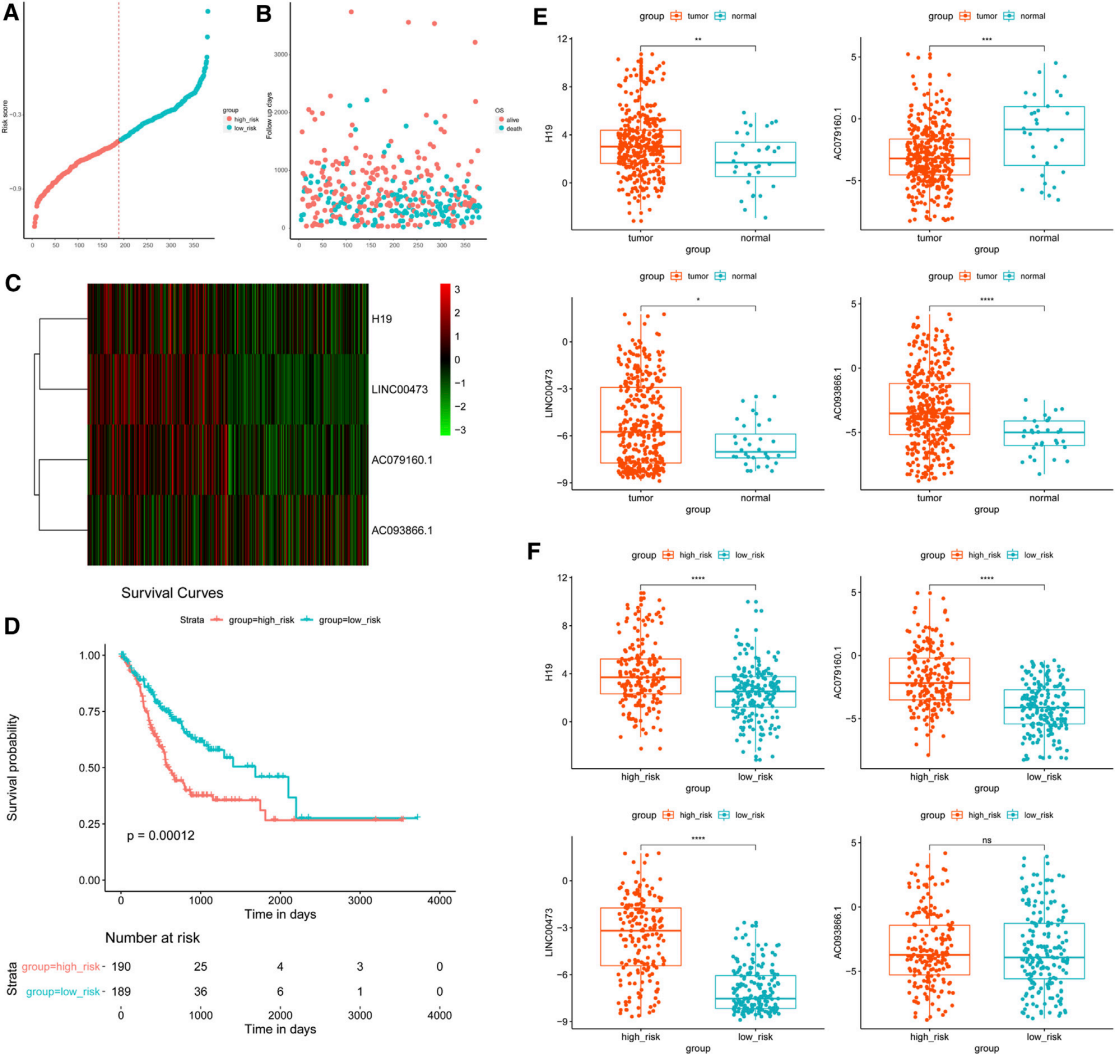
Risk score investigation of the differentially expressed long noncoding RNA (lncRNA) signature in gastric cancer. A risk score is shown according to the survival status and disease duration of the GC patients. **(A)** The abscissa refers to sample number and the ordinate depicts the risk score value. **(B)** GC patients were categorized into low-risk and high-risk groups based on the median inflection point of the risk score curve, which is represented by the dotted line. **(C)** lncRNA expression of low- and high-risk score patients. Colors from green to red indicate low to high lncRNA expression. **(D)** Overall survival (OS) status according to Kaplan–Meier analysis of the risk score. **(E, F)** Expression of the selected lncRNAs in **(E)** tumor vs. control samples, and **(F)** high-vs. low-risk patients. *p < 0.05, **p < 0.01, ***p < 0.001, ****p < 0.0001. NS, non-significant.

To evaluate the prognostic value of lncRNAs in GC, multivariate Cox proportional hazards regression analysis was conducted to build the lncRNA signature. The clinical data of 379 samples were used in the subsequent analysis. The findings disclosed that among all the lncRNAs, only H19, LINC00473, AC079160.1, and AC093866.1 had significant prognostic value in GC (Table 1 and Figure 5).

**TABLE 1:** Potential genes significantly associated with gastric cancer prognosis.

Gene name	Description	Ensembl ID
*AC079160.1*	Novel transcript	ENSG00000250546
*AC093866.1*	Novel transcript	ENSG00000251095
*Linc00473*	Long intergenic non-protein coding RNA473	90632(Gene ID)
*H19*	H19 imprinted maternally expressed transcript	ENSG00000130600

Accordingly, GC cases were grouped into high-risk (risk score ≥ −0.51, *n* = 190) and low-risk (risk score < −0.51, *n* = 189) categories. Next, survival analysis was performed based on the risk score, which revealed that patients with low-risk scores had a better overall prognosis compared with the high-risk score group (*p* = 0.00012). Additionally, the risk score overtly predicted the 5-year OS in GC (Figures 5A–D). The expression patterns of the four differently expressed lncRNAs in GC and nearby noncancerous tissues, as well as the groups with low- and high-risk scores, are shown in Figures 5E,F.

### 3.5 Univariable and Multivariable Analyses of Clinical Parameters

To investigate the association between the lncRNA signature and the survival status of the patients, several factors including sex, age, lymph node involvement, tumor stage, radiotherapy, and risk score were used for univariable and multivariable Cox regression analyses.

The multivariable analysis revealed that age [hazard ratio (HR) = 1.716, confidence interval (CI): 1.188–2.479; *p* = 0.004], tumor stage (HR = 1.783, 95% CI: 1.359–2.339, *p* < 0.001), radiation therapy (HR = 0.370, 95% CI: 0.219–0.623, *p* < 0.001), and risk score (HR = 1.987, 95% CI: 1.382–2.856, *p* < 0.001) were independent predictive factors (*p* < 0.001; Table 2).

**TABLE 2:** Prognostic potential of clinical features and the lncRNA risk score in gastric cancer.

Characteristics	Patients (*n*)	Univariate analysis		Multivariate analysis	
		HR (95% CI)	*p*-value	HR (95% CI)	*p*-value
Age	379	1.178 (1.178–2.258)	0.003	1.716 (1.188–2.479)	0.004
Sex	379	0.882 (0.882–1.755)	0.214	1.439 (0.974–2.125)	0.068
Lymph node involvement	379	0.572 (0.572–1.130)	0.209	0.761 (0.525–1.104)	0.151
Tumor stage	379	1.272 (1.272–2.053)	<0.001	1.783 (1.359–2.339)	<0.001
Radiation therapy	379	0.267 (0.267–0.713)	0.001	0.370 (0.219–0.623)	<0.001
Risk score	379	1.356 (1.356–2.614)	<0.001	1.987 (1.382–2.856)	<0.001

CI, confidence interval; HR, hazard ratio.

### 3.6 Expression of the Four lncRNAs Correlated With Patient Prognosis

**FIGURE 6:**
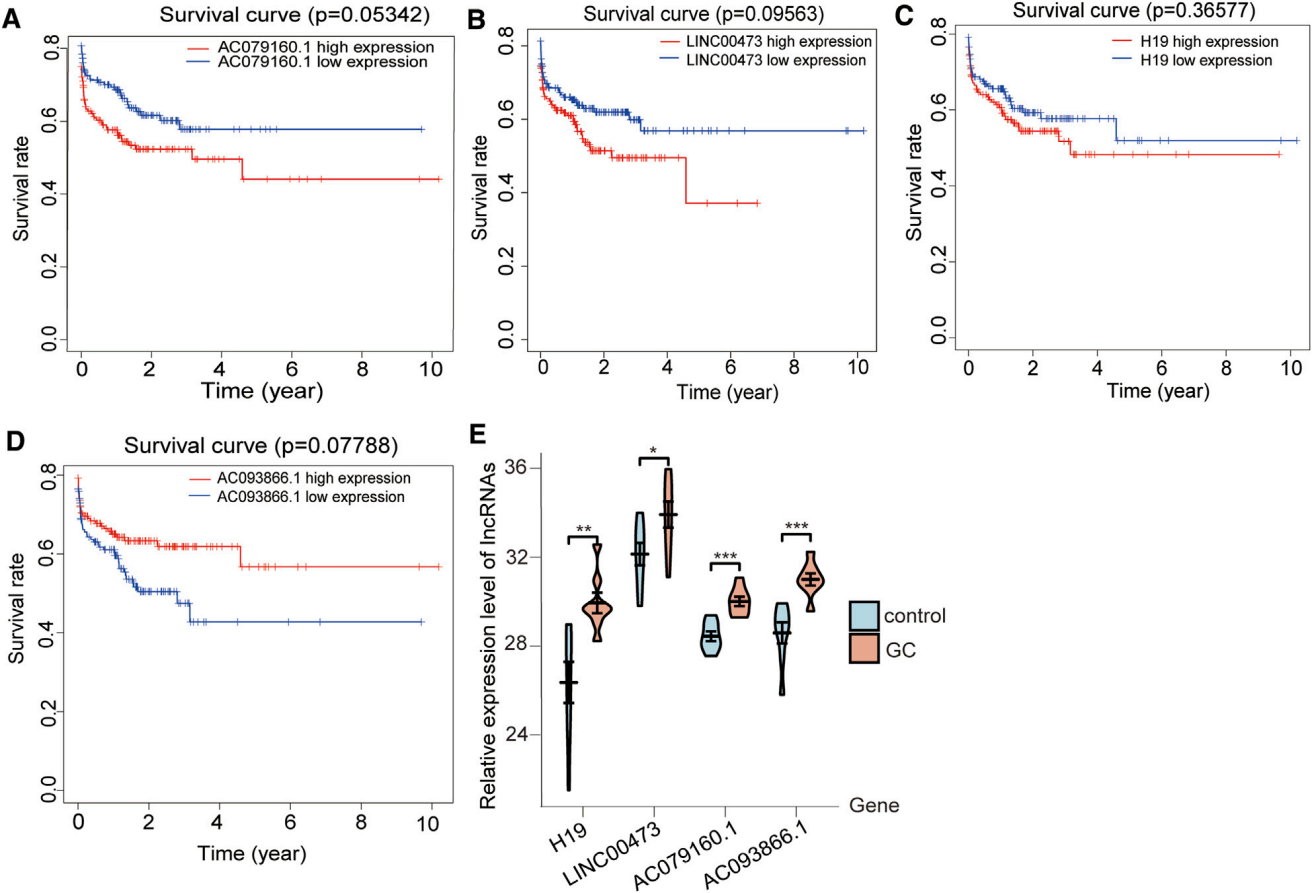
Expression of the four lncRNAs correlates with prognosis in GC. The levels of the lncRNAs **(A)** H19, **(B)** INC00473, and **(C)** AC079160.1 were positively correlated with patient survival, whereas **(D)** AC093866.1 showed a negative correlation with patient survival. Kaplan–Meier survival curves for GC patients were grouped into high and low risk based on the lncRNA expression signature. The *x*-axis represents overall survival time in years, while the survival rate is shown on the *y*-axis. *p*-values were determined by the log-rank test. **(E)** lncRNA expression evaluated by qRT-PCR in GC and adjoining noncancerous tissues. Data are presented as mean ± standard errors of the mean (*p < 0.05, **p < 0.01, ***p < 0.001).

The expression of the lncRNAs H19, INC00473, and AC079160.1 was positively correlated with prognosis (Figures 6A–C), whereas AC093866.1 was found to be negatively correlated with prognosis, as the risk coefficient became smaller with higher expression (Figure 6D). qRT-PCR was performed in eight groups of GC samples and adjacent noncancerous tissue samples to further verify the differential expression of the four lncRNAs (Supplementary Table S5). Kaplan–Meier survival curves for patients with GC were classified as high and low risk using the identified lncRNA expression signature. The elevated expression values of AC079160.1 (*p* < 0.001), AC093866.1 (*p* < 0.001), lnc00473 (*p* < 0.05), and H19 (*p* < 0.01) in GC tissue specimens were confirmed (Table 1 and Figure 6E).

### 3.7 External Validation of the Prognostic Value of the Four lncRNA Signature

**FIGURE 7:**
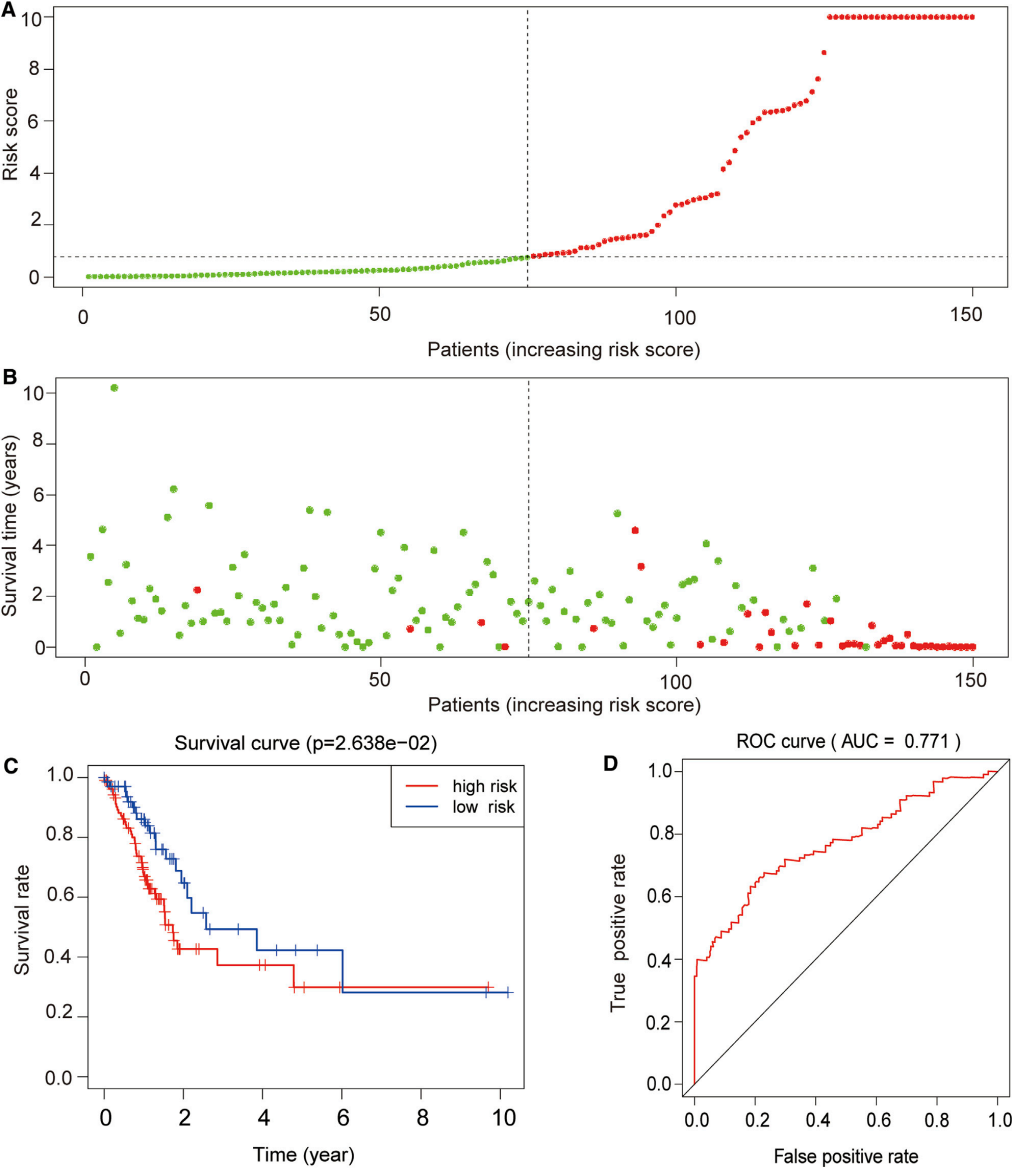
External validation of the prognostic value of the four lncRNA signatures. **(A)** Risk curve and (**B)** survival status chart of the validation dataset based on the proposed prognostic model. Each dot represents an individual patient, and the *y*-axis indicates the respective risk score. Green and red colors indicate the low- and high-risk groups, respectively. **(C)** Kaplan–Meier survival and **(D)** receiver operating characteristic (ROC) curves of the validation dataset based on the proposed prognostic model. The *x*-axis represents the overall survival time in years, and the *y*-axis indicates the survival rate of the patients according to their defined risk group. *p*-values were calculated by the log-rank test. Analysis of the area under the ROC curve (AUC = 0.771) indicated that the proposed model had a good risk prediction potential.

We successfully used a publicly available dataset (GSE62254, *n* = 300) to corroborate the reliability of the four lncRNA signatures. To assess the prognostic specificity and sensitivity potential of the proposed model, the receiver operating characteristic (ROC) curve analysis was performed, which showed that patients exhibiting high-risk scores displayed a significantly inferior OS than patients having low-risk scores (*p* < 0.0001; Figures 7A,B). The Kaplan–Meier survival analysis further showed that the survival rate in the high-risk group was significantly lower than that in the low-risk group (*p* < 0.05; Figure 7C). Similarly, the area under the curve (AUC) corresponding to the 5-year survival was found to be 0.771 (95% CI = 0.682–0.813, *p* < 0.0001), demonstrating the good prognostic capacity of the model (Figure 7D).

## 4 Discussion

GC seriously threatens the health and life of Chinese individuals, with high incidence, low early diagnosis rate, and low survival rate (Ferlay et al., 2015). Hence, it has rapidly become a highly important subject area to recognize early diagnostic and prognostic markers in GC. Recently, compelling evidence showed that lncRNAs have a major role in tumor growth and development. Nevertheless, there is limited knowledge regarding the relationship between GC and lncRNAs, thereby making it an open research area. Zhang *et al.* demonstrated that an abnormal histone modification leads to the activation of the lncRNA HOXC-AS3, which might contribute to the oncogenesis of GC (Zhang et al., 2018). Similarly, Zheng *et al.* revealed that KRT19P3, another lncRNA, potentially inhibits the growth of tumors *via* COPS7A-mediated NF-κB signals, implying its possible role for GC treatment (Zheng et al., 2019).

In the present study, 1,464 mRNAs, 14,376 lncRNAs, and 73 miRNAs were found to be differentially expressed in GC. GO function and KEGG pathway analyses led us to identify that the DERNAs were mostly involved in neuroactive ligand–receptor interaction, chemical carcinogenesis, epidermis development, and digestion, which are correlated with GC (Song et al., 2017). Overall, 910 differentially expressed lncRNAs were identified and included in the ceRNA network herein designed. Univariable and multivariable Cox regression analyses revealed statistically significant differences with respect to age, cancer stage, radiotherapy, and risk score groups, which were indeed independent predictors of prognosis. Four lncRNAs (lnc00473, H19, AC079160.1, and AC093866.1) were identified as prognostic biomarkers. Thus, we established a lncRNAs-based multi-molecular prognostic model to screen high-risk patients at high risk of poor prognosis.

Several lncRNAs are known to significantly affect cancer development and patient prognosis. Previous findings have reported that the aforementioned four lncRNAs are highly expressed in GC versus adjacent noncancerous tissues (Yang et al., 2012). In particular, H19 (Li et al., 2019) and lnc00473 (Zhang and Song, 2018) were shown to be highly expressed in GC. It was shown that H19 depletion activates AMPKα by downregulating H19 expression and inhibits MMP9, thereby playing an inhibitory role in GC (Li et al., 2019). In addition, overexpression of H19 in GC cells and tissues has been linked to tumor progression. Furthermore, H19 levels are negatively associated with miR-141 expression in GC cells, with H19 promoting malignancy while miR-141 plays an inhibitory role in human cancer cells (Zhou et al., 2015b; Yan et al., 2017). Lnc00473 can be isolated from the nucleus, and copolymerized and crosslinked with mitochondria and lipoproteins. Aberrant regulation of lnc00473 induces mutual interactions of lipolysis, respiration, and gene transcription related to mitochondrial oxidative metabolism. For example, lnc00473 is negatively expressed in the prefrontal cortex of dejected women. Zhang et al. found that lnc00473 is highly expressed in GC, also indicating a poorer prognostic outcome. Moreover, it was also reported that lnc00473 amounts are relatively higher in esophageal squamous cell carcinoma (ESCC), but its inhibition could suppress the malignant potential of ESCC cells (Zhang and Song, 2018; He, 2019; Issler et al., 2020; Tran et al., 2020). However, few studies have assessed AC079160.1 and AC093866.1 to date, and further investigations into their role in cancer are still needed.

In conclusion, H19, lnc00473, AC079160.1, and AC093866.1 may serve as valuable independent prognostic biomarkers, and pave the way for individualized diagnosis and treatment of GC. Herein, we established a prognostic model based on these four molecules, which may contribute to the identification of GC patients with the risk of poor prognosis. These discoveries deliver a potential foundation for the targeted treatment of GC. Further research is warranted to explore the underlying molecular mechanisms of these lncRNAs in GC, as well as larger sample-based clinical investigations to verify the present findings.

## Data Availability

The raw data supporting the conclusion of this article will be made available by the authors, without undue reservation.
