# Supplementary material for: Identification and Validation of a Potent Multi-lncRNA Molecular Model for Predicting Gastric Cancer Prognosis
Source: Front Genet. 2021 Dec 20;12:607748. doi: 10.3389/fgene.2021.607748 (PMC8720998; doi:10.3389/fgene.2021.607748)
Supplement: Supplementary file 1 [file DataSheet2.PDF]

Supplementary Table 3: The sequences of PCR primers.

Primers	Sequences	Size(bp)
AC079160.1	Forward: 5′-CCCTATTTGCCTGGGTATCAC -3′	104
	Reverse: 5′-GGCATCTGGCAGGTGACT-3′	
AC093866.1	Forward: 5′-GCCACCTAAGTGTCCATCAAC -3′	72
	Forward: 5′-GGCTGAATAGCACTCCGTTGT -3′	
Lnc00473	Forward: 5′-CTGAATGCAAAGCGGACACCTA-3′	79
	Reverse: 5′-CGTCCCCAAAACTGAGCACATA-3′	
H19(Human)	Forward: 5′-GGAATCGGCTCTGGAAGGT-3′	74
	Reverse: 5′-CTGTTCCGATGGTGTCTTTGATGTT-3′	
GAPDH (Human)	Forward: 5′-CCATGACAACTTTGGTATCGTGGAA -3′	107
	Reverse: 5′-GGCCATCACGCCACAGTTTC-3′	
