# Supplementary material for: Identification and Validation of a Potent Multi-lncRNA Molecular Model for Predicting Gastric Cancer Prognosis
Source: Front Genet. 2021 Dec 20;12:607748. doi: 10.3389/fgene.2021.607748 (PMC8720998; doi:10.3389/fgene.2021.607748)
Supplement: Supplementary file 3 [file DataSheet3.PDF]

1 Human GAPDH

sample number	Well	Fluor	Target	Content	Sample	Cq	Cq Mean	Cq Std. Dev
1a	A01	SYBR	GAPDH	Unkn-001	1a	14.27	14.206666667	0.06
	A02	SYBR	GAPDH	Unkn-001	1a	14.16	14.206666667	0.06
	A03	SYBR	GAPDH	Unkn-001	1a	14.19	14.206666667	0.06
1b	A04	SYBR	GAPDH	Unkn-002	1b	14.11	14.213333333	0.14
	A05	SYBR	GAPDH	Unkn-002	1b	14.16	14.213333333	0.14
	A06	SYBR	GAPDH	Unkn-002	1b	14.37	14.213333333	0.14
2a	A07	SYBR	GAPDH	Unkn-003	2a	13.72	13.716666667	0.03
	A08	SYBR	GAPDH	Unkn-003	2a	13.69	13.716666667	0.03
	A09	SYBR	GAPDH	Unkn-003	2a	13.74	13.716666667	0.03
2b	A10	SYBR	GAPDH	Unkn-004	2b	13.96	13.94	0.08
	A11	SYBR	GAPDH	Unkn-004	2b	14.01	13.94	0.08
	A12	SYBR	GAPDH	Unkn-004	2b	13.85	13.94	0.08
3a	A13	SYBR	GAPDH	Unkn-005	3a	13.83	13.77	0.07
	A14	SYBR	GAPDH	Unkn-005	3a	13.69	13.77	0.07
	A15	SYBR	GAPDH	Unkn-005	3a	13.79	13.77	0.07
3b	A16	SYBR	GAPDH	Unkn-006	3b	14.35	14.336666667	0.11
	A17	SYBR	GAPDH	Unkn-006	3b	14.44	14.336666667	0.11
	A18	SYBR	GAPDH	Unkn-006	3b	14.22	14.336666667	0.11
4a	A19	SYBR	GAPDH	Unkn-007	4a	13.82	13.826666667	0.06
	A20	SYBR	GAPDH	Unkn-007	4a	13.89	13.826666667	0.06
	A21	SYBR	GAPDH	Unkn-007	4a	13.77	13.826666667	0.06
4b	A22	SYBR	GAPDH	Unkn-008	4b	13.82	13.93	0.1
	A23	SYBR	GAPDH	Unkn-008	4b	13.95	13.93	0.1
	A24	SYBR	GAPDH	Unkn-008	4b	14.02	13.93	0.1
5a	B01	SYBR	GAPDH	Unkn-009	5a	13.59	13.6	0.01
	B02	SYBR	GAPDH	Unkn-009	5a	13.6	13.6	0.01
	B03	SYBR	GAPDH	Unkn-009	5a	13.61	13.6	0.01
5b	B04	SYBR	GAPDH	Unkn-010	5b	14.08	14.16	0.09
	B05	SYBR	GAPDH	Unkn-010	5b	14.15	14.16	0.09
	B06	SYBR	GAPDH	Unkn-010	5b	14.25	14.16	0.09
6a	B07	SYBR	GAPDH	Unkn-011	6a	13.84	13.94	0.1
	B08	SYBR	GAPDH	Unkn-011	6a	13.95	13.94	0.1
	B09	SYBR	GAPDH	Unkn-011	6a	14.03	13.94	0.1
6b	B10	SYBR	GAPDH	Unkn-012	6b	14.32	14.356666667	0.05
	B11	SYBR	GAPDH	Unkn-012	6b	14.41	14.356666667	0.05
	B12	SYBR	GAPDH	Unkn-012	6b	14.34	14.356666667	0.05
7a	B13	SYBR	GAPDH	Unkn-013	7a	13.59	13.56	0.12
	B14	SYBR	GAPDH	Unkn-013	7a	13.66	13.56	0.12
	B15	SYBR	GAPDH	Unkn-013	7a	13.43	13.56	0.12

7b	B16	SYBR	GAPDH	Unkn-014	7b	15.54	15.446666667	0.1
	B17	SYBR	GAPDH	Unkn-014	7b	15.35	15.446666667	0.1
	B18	SYBR	GAPDH	Unkn-014	7b	15.45	15.446666667	0.1
8a	B19	SYBR	GAPDH	Unkn-015	8a	13.39	13.353333333	0.05
	B20	SYBR	GAPDH	Unkn-015	8a	13.37	13.353333333	0.05
	B21	SYBR	GAPDH	Unkn-015	8a	13.3	13.353333333	0.05
8b	B22	SYBR	GAPDH	Unkn-016	8b	14.15	14.213333333	0.08
	B23	SYBR	GAPDH	Unkn-016	8b	14.19	14.213333333	0.08
	B24	SYBR	GAPDH	Unkn-016	8b	14.3	14.213333333	0.08

2 Human H19

sample number	Well	Fluor	Target	Content	Sample	Cq	Cq Mean	Cq Std. Dev
1a	C01	SYBR	H19	Unkn-017	1a	29.03	28.966666667	0.06
	C02	SYBR	H19	Unkn-017	1a	28.93	28.966666667	0.06
	C03	SYBR	H19	Unkn-017	1a	28.94	28.966666667	0.06
1b	C04	SYBR	H19	Unkn-018	1b	29.56	29.646666667	0.12
	C05	SYBR	H19	Unkn-018	1b	29.6	29.646666667	0.12
	C06	SYBR	H19	Unkn-018	1b	29.78	29.646666667	0.12
2a	C07	SYBR	H19	Unkn-019	2a	28.48	28.5	0.03
	C08	SYBR	H19	Unkn-019	2a	28.48	28.5	0.03
	C09	SYBR	H19	Unkn-019	2a	28.54	28.5	0.03
2b	C10	SYBR	H19	Unkn-020	2b	32.6	32.563333333	0.09
	C11	SYBR	H19	Unkn-020	2b	32.63	32.563333333	0.09
	C12	SYBR	H19	Unkn-020	2b	32.46	32.563333333	0.09
3a	C13	SYBR	H19	Unkn-021	3a	25.79	25.716666667	0.09
	C14	SYBR	H19	Unkn-021	3a	25.62	25.716666667	0.09
	C15	SYBR	H19	Unkn-021	3a	25.74	25.716666667	0.09
3b	C16	SYBR	H19	Unkn-022	3b	28.24	28.226666667	0.13
	C17	SYBR	H19	Unkn-022	3b	28.35	28.226666667	0.13
	C18	SYBR	H19	Unkn-022	3b	28.09	28.226666667	0.13
4a	C19	SYBR	H19	Unkn-023	4a	28.17	28.193333333	0.07
	C20	SYBR	H19	Unkn-023	4a	28.27	28.193333333	0.07
	C21	SYBR	H19	Unkn-023	4a	28.14	28.193333333	0.07
4b	C22	SYBR	H19	Unkn-024	4b	29.64	29.746666667	0.09
	C23	SYBR	H19	Unkn-024	4b	29.78	29.746666667	0.09
	C24	SYBR	H19	Unkn-024	4b	29.82	29.746666667	0.09
5a	D01	SYBR	H19	Unkn-025	5a	21.49	21.516666667	0.03
	D02	SYBR	H19	Unkn-025	5a	21.54	21.516666667	0.03
	D03	SYBR	H19	Unkn-025	5a	21.52	21.516666667	0.03
5b	D04	SYBR	H19	Unkn-026	5b	29.55	29.623333333	0.08

	D05	SYBR	H19	Unkn-026	5b	29.61	29.623333333	0.08
	D06	SYBR	H19	Unkn-026	5b	29.71	29.623333333	0.08
6a	D07	SYBR	H19	Unkn-027	6a	25.26	25.363333333	0.11
	D08	SYBR	H19	Unkn-027	6a	25.36	25.363333333	0.11
	D09	SYBR	H19	Unkn-027	6a	25.47	25.363333333	0.11
6b	D10	SYBR	H19	Unkn-028	6b	29.01	29.07	0.05
	D11	SYBR	H19	Unkn-028	6b	29.11	29.07	0.05
	D12	SYBR	H19	Unkn-028	6b	29.09	29.07	0.05
7a	D13	SYBR	H19	Unkn-029	7a	28.37	28.33	0.12
	D14	SYBR	H19	Unkn-029	7a	28.42	28.33	0.12
	D15	SYBR	H19	Unkn-029	7a	28.2	28.33	0.12
7b	D16	SYBR	H19	Unkn-030	7b	31.01	30.936666667	0.09
	D17	SYBR	H19	Unkn-030	7b	30.84	30.936666667	0.09
	D18	SYBR	H19	Unkn-030	7b	30.96	30.936666667	0.09
8a	D19	SYBR	H19	Unkn-031	8a	24.32	24.306666667	0.04
	D20	SYBR	H19	Unkn-031	8a	24.34	24.306666667	0.04
	D21	SYBR	H19	Unkn-031	8a	24.26	24.306666667	0.04
8b	D22	SYBR	H19	Unkn-032	8b	29.62	29.696666667	0.09
	D23	SYBR	H19	Unkn-032	8b	29.68	29.696666667	0.09
	D24	SYBR	H19	Unkn-032	8b	29.79	29.696666667	0.09

3 Human Linc00473

sample number	Well	Fluor	Target	Content	Sample	Cq	Cq Mean	Cq Std. Dev
1a	E01	SYBR	Licn00473	Unkn-033	1a	29.87	29.806666667	0.06
	E02	SYBR	Licn00473	Unkn-033	1a	29.75	29.806666667	0.06
	E03	SYBR	Licn00473	Unkn-033	1a	29.8	29.806666667	0.06
1b	E04	SYBR	Licn00473	Unkn-034	1b	31.03	31.11	0.11
	E05	SYBR	Licn00473	Unkn-034	1b	31.06	31.11	0.11
	E06	SYBR	Licn00473	Unkn-034	1b	31.24	31.11	0.11
2a	E07	SYBR	Licn00473	Unkn-035	2a	30.59	30.606666667	0.05
	E08	SYBR	Licn00473	Unkn-035	2a	30.57	30.606666667	0.05
	E09	SYBR	Licn00473	Unkn-035	2a	30.66	30.606666667	0.05
2b	E10	SYBR	Licn00473	Unkn-036	2b	32.46	32.443333333	0.11
	E11	SYBR	Licn00473	Unkn-036	2b	32.54	32.443333333	0.11
	E12	SYBR	Licn00473	Unkn-036	2b	32.33	32.443333333	0.11
3a	E13	SYBR	Licn00473	Unkn-037	3a	32.4	32.34	0.07
	E14	SYBR	Licn00473	Unkn-037	3a	32.27	32.34	0.07
	E15	SYBR	Licn00473	Unkn-037	3a	32.35	32.34	0.07

3b	E16	SYBR	Licn00473	Unkn-038	3b	33.8	33.77	0.1
	E17	SYBR	Licn00473	Unkn-038	3b	33.85	33.77	0.1
	E18	SYBR	Licn00473	Unkn-038	3b	33.66	33.77	0.1
4a	E19	SYBR	Licn00473	Unkn-039	4a	33.43	33.456666667	0.07
	E20	SYBR	Licn00473	Unkn-039	4a	33.54	33.456666667	0.07
	E21	SYBR	Licn00473	Unkn-039	4a	33.4	33.456666667	0.07
4b	E22	SYBR	Licn00473	Unkn-040	4b	35.46	35.546666667	0.09
	E23	SYBR	Licn00473	Unkn-040	4b	35.54	35.546666667	0.09
	E24	SYBR	Licn00473	Unkn-040	4b	35.64	35.546666667	0.09
5a	F01	SYBR	Licn00473	Unkn-041	5a	31.61	31.633333333	0.02
	F02	SYBR	Licn00473	Unkn-041	5a	31.65	31.633333333	0.02
	F03	SYBR	Licn00473	Unkn-041	5a	31.64	31.633333333	0.02
5b	F04	SYBR	Licn00473	Unkn-042	5b	35.02	35.123333333	0.11
	F05	SYBR	Licn00473	Unkn-042	5b	35.12	35.123333333	0.11
	F06	SYBR	Licn00473	Unkn-042	5b	35.23	35.123333333	0.11
6a	F07	SYBR	Licn00473	Unkn-043	6a	31.99	32.096666667	0.09
	F08	SYBR	Licn00473	Unkn-043	6a	32.13	32.096666667	0.09
	F09	SYBR	Licn00473	Unkn-043	6a	32.17	32.096666667	0.09
6b	F10	SYBR	Licn00473	Unkn-044	6b	33.01	33.043333333	0.03
	F11	SYBR	Licn00473	Unkn-044	6b	33.07	33.043333333	0.03
	F12	SYBR	Licn00473	Unkn-044	6b	33.05	33.043333333	0.03
7a	F13	SYBR	Licn00473	Unkn-045	7a	34	33.99	0.14
	F14	SYBR	Licn00473	Unkn-045	7a	34.12	33.99	0.14
	F15	SYBR	Licn00473	Unkn-045	7a	33.85	33.99	0.14
7b	F16	SYBR	Licn00473	Unkn-046	7b	36.08	35.956666667	0.12
	F17	SYBR	Licn00473	Unkn-046	7b	35.85	35.956666667	0.12
	F18	SYBR	Licn00473	Unkn-046	7b	35.94	35.956666667	0.12
8a	F19	SYBR	Licn00473	Unkn-047	8a	33.18	33.16	0.05
	F20	SYBR	Licn00473	Unkn-047	8a	33.2	33.16	0.05
	F21	SYBR	Licn00473	Unkn-047	8a	33.1	33.16	0.05
8b	F22	SYBR	Licn00473	Unkn-048	8b	34.28	34.336666667	0.08
	F23	SYBR	Licn00473	Unkn-048	8b	34.3	34.336666667	0.08
	F24	SYBR	Licn00473	Unkn-048	8b	34.43	34.336666667	0.08

4 Human AC079160.1

sample number	Well	Fluor	Target	Content	Sample	Cq	Cq Mean	Cq Std. Dev
1a	I01	SYBR	AC079160.1	Unkn-065	1a	28.13	28.076666667	0.05
	I02	SYBR	AC079160.1	Unkn-065	1a	28.03	28.076666667	0.05
	I03	SYBR	AC079160.1	Unkn-065	1a	28.07	28.076666667	0.05
1b	I04	SYBR	AC079160.1	Unkn-066	1b	30.02	30.136666667	0.15

	I05	SYBR	AC079160.1	Unkn-066	1b	30.08	30.136666667	0.15
	I06	SYBR	AC079160.1	Unkn-066	1b	30.31	30.136666667	0.15
2a	I07	SYBR	AC079160.1	Unkn-067	2a	29.1	29.086666667	0.01
	I08	SYBR	AC079160.1	Unkn-067	2a	29.08	29.086666667	0.01
	I09	SYBR	AC079160.1	Unkn-067	2a	29.08	29.086666667	0.01
2b	I10	SYBR	AC079160.1	Unkn-068	2b	29.39	29.36	0.06
	I11	SYBR	AC079160.1	Unkn-068	2b	29.4	29.36	0.06
	I12	SYBR	AC079160.1	Unkn-068	2b	29.29	29.36	0.06
3a	I13	SYBR	AC079160.1	Unkn-069	3a	28.19	28.116666667	0.08
	I14	SYBR	AC079160.1	Unkn-069	3a	28.04	28.116666667	0.08
	I15	SYBR	AC079160.1	Unkn-069	3a	28.12	28.116666667	0.08
3b	I16	SYBR	AC079160.1	Unkn-070	3b	30.14	30.113333333	0.1
	I17	SYBR	AC079160.1	Unkn-070	3b	30.2	30.113333333	0.1
	I18	SYBR	AC079160.1	Unkn-070	3b	30	30.113333333	0.1
4a	I19	SYBR	AC079160.1	Unkn-071	4a	29.38	29.376666667	0.08
	I20	SYBR	AC079160.1	Unkn-071	4a	29.45	29.376666667	0.08
	I21	SYBR	AC079160.1	Unkn-071	4a	29.3	29.376666667	0.08
4b	I22	SYBR	AC079160.1	Unkn-072	4b	29.2	29.29	0.08
	I23	SYBR	AC079160.1	Unkn-072	4b	29.31	29.29	0.08
	I24	SYBR	AC079160.1	Unkn-072	4b	29.36	29.29	0.08
5a	J01	SYBR	AC079160.1	Unkn-073	5a	28.44	28.46	0.02
	J02	SYBR	AC079160.1	Unkn-073	5a	28.46	28.46	0.02
	J03	SYBR	AC079160.1	Unkn-073	5a	28.48	28.46	0.02
5b	J04	SYBR	AC079160.1	Unkn-074	5b	29.85	29.956666667	0.1
	J05	SYBR	AC079160.1	Unkn-074	5b	29.97	29.956666667	0.1
	J06	SYBR	AC079160.1	Unkn-074	5b	30.05	29.956666667	0.1
6a	J07	SYBR	AC079160.1	Unkn-075	6a	28.78	28.876666667	0.09
	J08	SYBR	AC079160.1	Unkn-075	6a	28.9	28.876666667	0.09
	J09	SYBR	AC079160.1	Unkn-075	6a	28.95	28.876666667	0.09
6b	J10	SYBR	AC079160.1	Unkn-076	6b	30.51	30.55	0.06
	J11	SYBR	AC079160.1	Unkn-076	6b	30.62	30.55	0.06
	J12	SYBR	AC079160.1	Unkn-076	6b	30.52	30.55	0.06
7a	J13	SYBR	AC079160.1	Unkn-077	7a	27.57	27.55	0.11
	J14	SYBR	AC079160.1	Unkn-077	7a	27.65	27.55	0.11
	J15	SYBR	AC079160.1	Unkn-077	7a	27.43	27.55	0.11
7b	J16	SYBR	AC079160.1	Unkn-078	7b	31.14	31.07	0.07
	J17	SYBR	AC079160.1	Unkn-078	7b	31	31.07	0.07
	J18	SYBR	AC079160.1	Unkn-078	7b	31.07	31.07	0.07
8a	J19	SYBR	AC079160.1	Unkn-079	8a	28	27.97	0.03
	J20	SYBR	AC079160.1	Unkn-079	8a	27.97	27.97	0.03
	J21	SYBR	AC079160.1	Unkn-079	8a	27.94	27.97	0.03
8b	J22	SYBR	AC079160.1	Unkn-080	8b	29.51	29.56	0.06
	J23	SYBR	AC079160.1	Unkn-080	8b	29.54	29.56	0.06

	J24	SYBR	AC079160.1	Unkn-080	8b	29.63	29.56	0.06

5 Human AC093866.1

sample number	Well	Fluor	Target	Content	Sample	Cq	Cq Mean	Cq Std. Dev
1a	G01	SYBR	AC093866.1	Unkn-049	1a	28.73	28.646666667	0.07
	G02	SYBR	AC093866.1	Unkn-049	1a	28.6	28.646666667	0.07
	G03	SYBR	AC093866.1	Unkn-049	1a	28.61	28.646666667	0.07
1b	G04	SYBR	AC093866.1	Unkn-050	1b	30.66	30.78	0.14
	G05	SYBR	AC093866.1	Unkn-050	1b	30.75	30.78	0.14
	G06	SYBR	AC093866.1	Unkn-050	1b	30.93	30.78	0.14
2a	G07	SYBR	AC093866.1	Unkn-051	2a	25.83	25.81	0.02
	G08	SYBR	AC093866.1	Unkn-051	2a	25.79	25.81	0.02
	G09	SYBR	AC093866.1	Unkn-051	2a	25.81	25.81	0.02
2b	G10	SYBR	AC093866.1	Unkn-052	2b	30.67	30.633333333	0.08
	G11	SYBR	AC093866.1	Unkn-052	2b	30.69	30.633333333	0.08
	G12	SYBR	AC093866.1	Unkn-052	2b	30.54	30.633333333	0.08
3a	G13	SYBR	AC093866.1	Unkn-053	3a	29.98	29.92	0.09
	G14	SYBR	AC093866.1	Unkn-053	3a	29.82	29.92	0.09
	G15	SYBR	AC093866.1	Unkn-053	3a	29.96	29.92	0.09
3b	G16	SYBR	AC093866.1	Unkn-054	3b	31.58	31.563333333	0.14
	G17	SYBR	AC093866.1	Unkn-054	3b	31.69	31.563333333	0.14
	G18	SYBR	AC093866.1	Unkn-054	3b	31.42	31.563333333	0.14
4a	G19	SYBR	AC093866.1	Unkn-055	4a	28.76	28.78	0.06
	G20	SYBR	AC093866.1	Unkn-055	4a	28.85	28.78	0.06
	G21	SYBR	AC093866.1	Unkn-055	4a	28.73	28.78	0.06
4b	G22	SYBR	AC093866.1	Unkn-056	4b	30.82	30.946666667	0.11
	G23	SYBR	AC093866.1	Unkn-056	4b	30.99	30.946666667	0.11
	G24	SYBR	AC093866.1	Unkn-056	4b	31.03	30.946666667	0.11
5a	H01	SYBR	AC093866.1	Unkn-057	5a	28.84	28.873333333	0.03
	H02	SYBR	AC093866.1	Unkn-057	5a	28.9	28.873333333	0.03
	H03	SYBR	AC093866.1	Unkn-057	5a	28.88	28.873333333	0.03
5b	H04	SYBR	AC093866.1	Unkn-058	5b	30.91	30.99	0.08
	H05	SYBR	AC093866.1	Unkn-058	5b	31	30.99	0.08
	H06	SYBR	AC093866.1	Unkn-058	5b	31.06	30.99	0.08
6a	H07	SYBR	AC093866.1	Unkn-059	6a	29.36	29.436666667	0.07
	H08	SYBR	AC093866.1	Unkn-059	6a	29.45	29.436666667	0.07
	H09	SYBR	AC093866.1	Unkn-059	6a	29.5	29.436666667	0.07
6b	H10	SYBR	AC093866.1	Unkn-060	6b	31.22	31.266666667	0.05
	H11	SYBR	AC093866.1	Unkn-060	6b	31.32	31.266666667	0.05

	H12	SYBR	AC093866.1	Unkn-060	6b	31.26	31.266666667	0.05
7a	H13	SYBR	AC093866.1	Unkn-061	7a	29.73	29.69	0.12
	H14	SYBR	AC093866.1	Unkn-061	7a	29.79	29.69	0.12
	H15	SYBR	AC093866.1	Unkn-061	7a	29.55	29.69	0.12
7b	H16	SYBR	AC093866.1	Unkn-062	7b	32.35	32.236666667	0.12
	H17	SYBR	AC093866.1	Unkn-062	7b	32.12	32.236666667	0.12
	H18	SYBR	AC093866.1	Unkn-062	7b	32.24	32.236666667	0.12
8a	H19	SYBR	AC093866.1	Unkn-063	8a	27.59	27.543333333	0.05
	H20	SYBR	AC093866.1	Unkn-063	8a	27.55	27.543333333	0.05
	H21	SYBR	AC093866.1	Unkn-063	8a	27.49	27.543333333	0.05
8b	H22	SYBR	AC093866.1	Unkn-064	8b	29.52	29.57	0.06
	H23	SYBR	AC093866.1	Unkn-064	8b	29.55	29.57	0.06
	H24	SYBR	AC093866.1	Unkn-064	8b	29.64	29.57	0.06
