# Supplementary material for: Identification and Validation of a Potent Multi-lncRNA Molecular Model for Predicting Gastric Cancer Prognosis
Source: Front Genet. 2021 Dec 20;12:607748. doi: 10.3389/fgene.2021.607748 (PMC8720998; doi:10.3389/fgene.2021.607748)
Supplement: Supplementary file 4 [file DataSheet1.PDF]

Supplemental table 1. Clinical data of the patients whose samples were assessed by qRT-PCR, representing 8 matched, paired gastric tumor–normal tissue samples.

Clinical Features	Variable	Tissue (All patients, n = 8)
Sex	Males	6
	Females	2
Age	Median, range	69, 42-85
Lauren staging	Intestinal gastric cancer	5
	Diffuse gastric cancer	3
Tumor location	Cardiac ostium	5
	Gastric antrum	2
	Angle of stomach	1
Her-2	Positive	3
	Negative	5
Relevant family history	Yes	0
	No	8
TNM	T1	1
	T2	1
	T3	6
	T4	0
	N0	2
	N1	3
	N2	2

	N3	1
	M0	8
	M1	0
AJCC staging	I	2
	II	0
	IIa	0
	IIb	3
	IIIa	2
	IIIb	1
	IIIc	0
	IV	0
Tumor grade	High differentiation	0
	Middle differentiation	3
	Low differentiation	3
	Signet ring cell carcinoma	2
Chemotherapy	No	8
	Yes	0
